# Application of short message service to control blood cholesterol: a field trial

**DOI:** 10.1186/s12911-017-0427-3

**Published:** 2017-03-28

**Authors:** Saeed Sadeghian, Mohsen Shams, Zahra Alipour, Soheil Saadat, Reza Hamidian, Maryam Shahrzad

**Affiliations:** 1 Tehran Heart Center, Tehran University of Medical Sciences, Tehran, Iran; 2 Social Determinants of Health Research Center, Yasuj University of Medical Sciences, Yasuj, Iran; 3 Sina Research Development Center, Tehran University of Medical Sciences, Tehran, Iran; 4 Sina Trauma and Surgery Research Center, Tehran University of Medical Sciences, Tehran, Iran; 5 Department of e-Health, Virtual School, Tehran University of Medical Sciences, Tehran, Iran

**Keywords:** Short message service, Blood cholesterol, Coronary disease

## Abstract

**Background:**

Despite recommendations, many middle-age adults neglect to check their blood cholesterol levels. Short message service (SMS, also known as texting) has been seldom studied for preventive education. We estimated how SMS can be a cost-effective method in encouraging people to check their blood cholesterol levels.

**Methods:**

In a field trial, 3600 cell phone users (age > 30) were randomly assigned to the intervention (N: 1200) and the control groups (N: 2400). An SMS was sent to the intervention group for five rounds every two weeks, which targeted the cognitive and affective learning and finally advised the blood cholesterol level to be checked, if not checked during the past twelve months. Two weeks after the last round, both groups were asked for the time/level of their latest blood cholesterol, family history of early cardiac death and having a family member with coronary heart disease (CHD), and to report their attitude about whether annual blood sampling is worth the cost and time to prevent CHD. Moreover, the intervention group was asked if they remembered the SMS content. The cost-effectiveness was evaluated by estimating the “number needed to treat” (NNT) and calculating the cost of sending SMS to that number of people.

**Results:**

In the intervention group, 629 individuals (72.0%) recalled the SMS content. The factors associated with cholesterol screening during the past two years were older age, diabetes, family history of coronary disease, higher education, female gender and being non-smoker. In both groups, women were significantly more aware of their blood cholesterol level (68.7% vs. 53.6%). The relative frequency of respondents who believed it was not worth checking their cholesterol annually was significantly lower in the intervention group (*P <* 0.001). The intervention group was significantly more likely to check its blood cholesterol levels (OR:1.22) after adjustment for age, diabetes, family history of CHD and smoking. The NNT was estimated ≈ 25 for the general population and ≈ 11 for those who received SMS and had a family member with CHD.

**Conclusions:**

We would postulate that SMS could affect people’s adherence to preventive programs. Relatives of patients admitted with a diagnosis of CHD should be prioritized for superior cost-effectiveness and logistical feasibility.

## Background

Coronary heart disease (CHD) is among the main causes of morbidity and mortality across the world; Iran is no exception [1, 2]. High cholesterol level is a major cause of disease burden in both the developed and developing countries, which roughly causes 2.6 million deaths (4.5% of total deaths) and 29.7 million disability-adjusted life years (DALYS) [3]. That is why a diagnosis of dyslipidemia is important, especially in individuals at higher risk of CHD, because dyslipidemia is associated with increased risk of cardiovascular events [4, 5]. Although the National Cholesterol Education Program Adult Treatment Panel III (NCEP ATP III) recommends starting universal screening for high cholesterol levels at the age of 20 [6], many adults between the ages 20 and 44 years have missed the opportunity to get their blood cholesterol levels checked in their past 5 years [4].

Some studies have demonstrated that a well-designed health education program can enhance people’s adherence to screening programs by means of raising their awareness and increasing their knowledge of health [7]. The past decade has witnessed the increasing popularity of cell phones in most countries. The vast majority of adult Iranians own cell phones and texting (by using short message service, or SMS) is a very common practice among cell phone users in Iran. Therefore, SMS could be utilized for educational interventions for a large segment of the population at any time or place. Simplicity, low cost, widespread use and instant delivery are the rewarding features of employing this particular method [8, 9].

SMS-related improvements in healthcare outcomes have been reported in form of improved outpatient clinic attendance, encouragement for weight loss, compliance with taking medication, growing support for diabetic and asthmatic patients, increased smoking quit rates and increased sunscreen application [8, 10]. However, most of these studies have focused on clinical care interventions in sick individuals and there has been a paucity of information in the domain of prevention in healthy individuals. As an example of the latter group, a recent study showed that a telemonitoring program via SMS was an effective strategy in the secondary prevention of acute coronary syndrome survivors [11]. Park et al. also reported improved control of blood pressure, body weight, waist circumference and HDL-C levels in patients with concomitant obesity and hypertension after eight weeks of SMS intervention [12].

In the present study, we studied the cost-effectiveness of SMS as a tool to encourage people to check their blood cholesterol levels. To the best of the authors’ knowledge, this is the first published study that focuses on the cost-effectiveness of SMS in encouraging people to control a CHD risk factor.

## Methods

### Study design

**Fig. 1:**
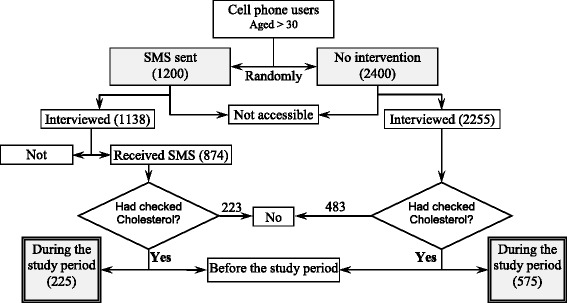
Study design for application of short message service (SMS) to control blood cholesterol: a field trial

An advertising company specializing in text commercials and surveys was hired to randomly select from its data pool three thousand and six hundred cell phone users who were older than thirty years old and residents of the Tehran Metropolitan Area in the year 2008. The company sent SMS to the participants and transferred their anonymized information to the authors. The study was a field trial, where participants were randomly assigned to either the intervention or the control group. The allocation ratio was 1:2 respectively (Fig. 1). An SMS package was sent to the intervention group for five rounds, every two weeks. At each round, the intervention group was sent a daily message on three consecutive days, beginning with the first day of the workweek. The message sent on the first day targeted the cognitive learning of the recipient, explaining that high blood cholesterol was a major risk factor of CHD. The second day’s message aimed toward the affective learning suggesting that it was worth checking blood cholesterol annually for the prevention of CHD development. The third day's message was a psychomotor-based approach that directly advised the recipient to get blood cholesterol level checked as soon as possible if (s)he was thirty years of age or older, especially if (s)he had not checked it during the prior twelve months. All the messages were subscribed by Tehran Heart Center as the sender and the length of each SMS was restricted to a single message.

Both groups were reached via a phone call two weeks after the dispatch of the last round of messages. Overall, 1138 (94.8%) and 2255 (94.0%) participants in the intervention and the control group responded, respectively. During the interview period, the participants remained anonymous. Participants were informed at the beginning of the interview that a) this was a research study gathering no identifying information, b) the participants have the right not to answer any question at their discretion, and c) the participants are free to end their contribution to the study at any time for any reason.

The respondents were asked when they had last checked their blood cholesterol levels, what its value was, whether or not they had a family history of early cardiac death and if they had a family member with CHD. Additionally, both groups’ demographics including age, sex, education level, occupation, history of smoking and status of diabetes mellitus were collected. The intervention group was asked if they had received any SMS from Tehran Heart Center and whether they can remember its content. The respondents were asked to report their attitude toward this statement: “Is annual blood sampling worth the cost and time to prevent CHD?”

### Statistical analysis

The data were analyzed using SPSS 13.0 software (IBM Corp., USA). The continuous data are shown as mean (± standard deviation [SD]). The chi-square test was used to compare the categorical data between the groups for univariable analysis, and a logistic regression model was employed for multi-variable analysis.

In the logistic regression model, “checking blood cholesterol during the study period” was considered as the dependent variable. “Receiving the educational text” was the independent variable of interest. The effects of age, being diabetic, a family history of CHD, higher education, and smoking on association of the dependent variable and the main independent variable were controlled by including them in the model.

The cost-effectiveness of the SMS intervention was evaluated by estimating the “number needed to treat” (NNT) and calculating the cost of sending SMS to that specific number of people. The NNT is the average number of subjects who need treatment to prevent one additional adverse outcome (for example, the number of patients that need treatment for its benefit compared with control subjects in a clinical trial). The NNT represents the inverse of absolute risk reduction. To estimate the NNT based on adjusted Odds Ratio (OR), the following formula was applied:

NNT = |(1-(PEER×(1-OR)))/((1-PEER) × (PEER) × (1-OR))|; where PEER is the prevalence of checking blood cholesterol levels during the study period in the unexposed (control) group.

## Results

The intervention and the control groups consisted of 1200 and 2400 cell phone users, respectively. The authors were unable to reach 205 participants (62 from the intervention and 145 from the control group) (Fig. 1). In the intervention group, 874 individuals (76.8%) confirmed that they had received the messages, and 629 persons (72.0%) correctly recalled the message content. The basic characteristics of the respondents are depicted in Table 1.

**Table 1:** Characteristics of study groups in the application of short message service (SMS) to control blood cholesterol: a field trial

Characteristics	Control (No SMS) Number (%)	Intervention (received SMS) Number (%)
Male	1380 (61.2)	680 (59.8)
Smoker	384 (17.0)	240 (21.1)
Diabetic	87 (3.9)	33 (3.0)
Family History		
CHD^a^	476 (21.1)	311 (27.3)
Early cardiac death	165 (7.3)	111 (9.8)
Education		
Illiterate	17 (0.8)	5 (0.4)
Up to middle school	245 (10.9)	125 (11.0)
Up to high school	450 (20.0)	311 (27.3)
Diploma	114 (5.1)	56 (4.9)
College	391 (17.3)	278 (24.4)
Bachelor or higher	192 (8.5)	106 (9.3)
Declined to respond	846 (37.5)	257 (22.6)
Age (year ± SD)^b^	40.15 (±12.42)	41.99 (±9.17)

^a^CHD, coronary heart disease; ^b^SD, standard deviation

Of all the study participants, 1322 individuals (36.7%) had not checked their blood cholesterol levels during the past two years. The variables associated with an absence of blood cholesterol screening during the past two years are presented in Table 2.

**Table 2:** Variables associated with absent blood cholesterol checks during two years prior to the study

Variables	Coefficient	P^c^	OR^d^
Receiving SMS^a^	−0.170	0.063	0.844
Older age	−0.052	<0.001	0.949
Diabetes mellitus	−0.686	0.001	0.504
Family history of CHD^b^	−0.240	0.010	0.786
Higher education	−0.202	<0.001	0.817
Sex (female vs. male)	−0.272	0.014	0.762
Smoking	0.496	<0.001	1.642
Constant	4.216		

^a^SMS, short message service; ^b^CHD, coronary heart disease; ^c^P, *P* value; ^d^OR, odds ratio

The proportion of people who had never checked their blood cholesterol levels was twice as much in men (27.0%) than in women (13.3%), which was statistically significant (*P <* 0.001). Table 3 demonstrates blood cholesterol levels in respondents who recalled it during the interview.

**Table 3:** The blood cholesterol levels (mean [±standard deviation]) in study participants who checked and recalled their blood cholesterol levels

Variable	Blood cholesterol level (mg/dl) Mean (± SD)	N
Sex		
Male	214.4 (±74.7)	163
Female	210.8 (±47.0)	61
DM^a^		
Yes	200.1 (±32.6)	13
No	214.3 (±69.6)	211
Smoking		
Yes	226.3 (±79.7)	47
No	209.3 (±64.1)	177

^a^DM, diabetes mellitus

Regardless of the study group, women were significantly more aware of their blood cholesterol levels than men were (68.7% vs. 53.6%, *P <* 0.001).

### Attitude

The relative frequency of the respondents who believed it was not worth checking their blood cholesterol levels annually in terms of the cost and time needed was 6.46% in the control group and 3.25% in the intervention group (*P <* 0.001).

### Practice

Eight hundred participants had checked their blood cholesterol levels during the intervention period (Fig. 1). In the multivariable analysis, the intervention group participants were more likely to check their blood cholesterol levels in comparison to the control participants (OR: 1.22) after adjustment for age, diabetes mellitus status, family history of CHD and history of smoking; the difference constituted statistical significance (Table 4).

**Table 4:** Variables associated with checking blood cholesterol levels during the intervention period

Variables	Coefficient	P^c^	OR^d^
Receiving SMS^a^	0.200	0.033	1.22
Older age	0.043	<0.001	1.04
Diabetes mellitus	0.754	<0.001	2.13
Family history of CHD^b^	0.248	0.009	1.28
Higher education	0.130	<0.001	1.14
Smoking	−0.418	<0.001	0.66
Constant	−3.987	<0.001	

^a^SMS, short message service; ^b^CHD, coronary heart disease; ^c^P, *P* value; ^d^OR, odds ratio

### Cost-effectiveness

The cost of using SMS was about 0.01 USD (United States Dollar) per message at the time of the study. Fifteen text messages were sent to every person in the intervention group; however, only 76.8% acknowledged the receipt of the messages during the interview period. Accordingly, the cost of a successful SMS delivery to a single person was 0.195 USD.

In the control group, 25.5% had checked their blood cholesterol levels. Therefore, the NNT can be calculated as (1-(0.255 × (1–1.22)))/((1–0.255) × (0.255) × (1–1.22)) ≈ 25 for the general population. The Odds Ratio (OR) for those who had received messages and had a family member with CHD was estimated to be e^(0.200+ 0.248)^ = 1.57, with the NNT being about 11 for this specific group.

## Discussion

Screening young adults for serum lipid disorders is recommended by numerous specialties, particularly young adults with CHD, CHD equivalents or individuals with one or more cardiac risk factors [13, 14]. Nevertheless, convincing healthy people to take action and check their blood lipid levels appears to be almost a farfetched achievement [10]. As found in our study, 39% of the respondents had not checked their blood cholesterol levels during the two-year period leading up to our study.

We used cellular texting (SMS) as a tool for the reminder strategy in order to increase knowledge and attitude toward the important role that cholesterol plays in developing CHD, as well as increasing healthy people’s adherence to a preventive behavior toward CHD. We noted that this strategy inspired one out of twenty-five people in the general population to actually check his/her blood cholesterol level. We also found that the NNT for this intervention was less for people with a positive family history of CHD; therefore, the effectiveness of this strategy could be enhanced by focusing on the relatives of CHD patients.

During the intervention period, the attitude of the intervention group toward checking their cholesterol levels, regardless of the required time and money, was improved and this result was in accordance with the improvement in the preventive practice of testing the blood cholesterol level. Improvement in healthcare outcomes has been reported in previous studies examining the use of SMS [8, 10]; however, when Cocosila et al. evaluated the usefulness of SMS, they found non-significant improvement in healthy people’s adherence to taking vitamin C [9].

In the context of health economics, the cost of motivating a person in the general population to actually check his/her blood cholesterol level was 4.9 (25_NNT_ × 0.195_cost of successful SMS delivery_) USD. The cost-effectiveness of this strategy could be augmented by restricting the target population to the relatives of high-risk CHD patients. With this restriction in mind, it would cost 2.1 USD per person to inspire a healthy relative of a high-risk CHD patient to do a screening for blood cholesterol level. A few studies in the existing literature have evaluated the cost-effectiveness of texting. Chen et al. [15] showed that reminding each patient of an appointment via SMS was significantly cheaper (0.31 Yuan per SMS) than telephone (0.48 Yuan per call). Koshy et al. [16] also used SMS reminders for ophthalmology outpatient appointments and concluded it was more cost-effective and less demanding than traditional methods.

The cost-effectiveness of the texting strategy also depends on the optimum number of messages, which demands further studies.

In the present study, female respondents were more aware of their cholesterol level and also checked their blood cholesterol levels twice as much as male participants. Considering the role of the male gender as an independent CHD risk factor [17], specific interventions are required, in order to propel men to enhance their health awareness. Furthermore, this result regarding gender as a factor can be interpreted as a sign of women having more sensitivity and attention to their health concerns [18].

In this study we randomly texted general population. Since higher blood cholesterol levels are expected in certain populations such as males, diabetics and smokers [19–21], stratification of the target group according to sex, existence of diabetes mellitus and smoking may yield better results. For this reason, these specific groups must be prioritized for screening programs.

During the study period, we addressed two important reasons for non-adherence within the participants: forgetfulness that was addressed by means of sending reminders every two weeks, and lack of knowledge, which was addressed by sending texts, which pointed the importance as well as one serious side effect of having high blood cholesterol levels [8].

In the light of our study results, we would postulate that SMS could affect people’s adherence to preventive programs for several reasons. As stated before, cell phones are widely used across Iran, and texting by using SMS is a common practice. Simplicity, efficiency, confidentiality [22], affordability and feasibility are also among the reasons why SMS use should be considered for preventive programs [8, 10].

In spite of these unique features of SMS, we should bear in mind that certain groups are less likely to access cell phones or be adequately educated to read texts. Those groups including, though not limited to, the unemployed, the destitute, the illiterate and the elderly need further education or assistance regarding the use of SMS. Another important limitation of this strategy was highlighted by our study; in the sense that the correct recollection of the message content was only 55.3% of the intervention group’s participants.

## Conclusions

In this study, we were able to document the effectiveness of utilizing SMS as a preventive strategy for CHD. However, further research is needed to determine the optimal number of SMS messages to be sent, the optimal interval between the messages, and the most efficacious content and wording of the messages when accounting for different populations (for instance, people with dissimilar occupations and educational background).

## Abbreviations

CHD: Coronary heart disease
DALYS: Disability-adjusted life years
DM: Diabetes mellitus
NNT: Number needed to treat
OR: Odds ratio
SD: Standard deviation
SMS: Short message service
USD: United States Dollar
